# A TFEB nuclear export signal integrates amino acid supply and glucose availability

**DOI:** 10.1038/s41467-018-04849-7

**Published:** 2018-07-11

**Authors:** Linxin Li, Hans J. Friedrichsen, Sarah Andrews, Sarah Picaud, Laurent Volpon, Kaochin Ngeow, Georgina Berridge, Roman Fischer, Katherine L. B. Borden, Panagis Filippakopoulos, Colin R. Goding

**Affiliations:** 1 Ludwig Institute for Cancer Research, Nuffield Department of Clinical Medicine, University of Oxford, Headington, Oxford, OX3 7DQ UK; 2 Structural Genomics Consortium, Nuffield Department of Clinical Medicine, University of Oxford, Headington, Oxford, OX3 7DQ UK; 3 Institute of Research in Immunology and Cancer (IRIC), Department of Pathology and Cell Biology, Université de Montréal, Pavillon Marcel-Coutou, Chemin de la Polytechnique, Montréal, QC H3T 1J4 Canada; 4 Discovery Proteomics Facility, Target Discovery Institute, Nuffield Department of Clinical Medicine, University of Oxford, Headington, Oxford, OX3 7FZ UK

## Abstract

How cells coordinate the response to fluctuating carbon and nitrogen availability required to maintain effective homeostasis is a key issue. Amino acid limitation that inactivates mTORC1 promotes de-phosphorylation and nuclear translocation of Transcription Factor EB (TFEB), a key transcriptional regulator of lysosome biogenesis and autophagy that is deregulated in cancer and neurodegeneration. Beyond its cytoplasmic sequestration, how TFEB phosphorylation regulates its nuclear-cytoplasmic shuttling, and whether TFEB can coordinate amino acid supply with glucose availability is poorly understood. Here we show that TFEB phosphorylation on S142 primes for GSK3β phosphorylation on S138, and that phosphorylation of both sites but not either alone activates a previously unrecognized nuclear export signal (NES). Importantly, GSK3β is inactivated by AKT in response to mTORC2 signaling triggered by glucose limitation. Remarkably therefore, the TFEB NES integrates carbon (glucose) and nitrogen (amino acid) availability by controlling TFEB flux through a nuclear import-export cycle.

## Abstract (web summary)

On amino acid deprivation TFEB translocates from the cytoplasm to the nucleus. Here the authors identify a nuclear export signal in TFEB that requires dual phosphorylation at the S142 ERK/mTORC1 and S138 GSK3β sites, and further show glucose limitation drives nuclear accumulation of TFEB and inhibits GSK3β via an mTORC2-AKT dependent mechanism.

## Introduction

On amino acid limitation TFEB translocates to the nucleus to promote lysosome biogenesis and autophagy^1–3^ that recycles unwanted organelles to increase amino acid availability. TFEB subcellular localization is controlled by the amino acid sensing mTORC1 complex^4,5^ that phosphorylates TFEB on S211 to enable cytoplasmic sequestration via 14-3-3 protein interaction^6^. Interaction of TFEB with the mTORC1-Rag GTPase-Ragulator complex is facilitated by TFEB phosphorylation on Ser3 by MAP4K3^7^, a kinase activated by amino acids^8–10^. Cytoplasmic localization is also promoted by mTORC1 and ERK2 phosphorylation on S142^1,11^, by mTOR phosphorylation on S122^12^, and by GSK3β phosphorylation on S138^13^. However, although GSK3β can activate mTORC1 signaling via phosphorylation of RAPTOR on S859^14^, GSK3β inhibition has been reported not to affect mTOR signaling^15^ and neither the physiological trigger for GSK3β phosphorylation, nor how S142 and S138 modification prevent TFEB nuclear accumulation are known.

In addition to promoting lysosome biogenesis in response to amino acid limitation, TFEB can also enhance the integrated stress response mediated by ATF4^16^ and acts as a nexus for nutrient sensing and resolution of any supply-demand disequilibrium. It is also a key effector of the beneficial effects of exercise by controlling metabolic flexibility in muscle^17^, protects against inflammation-mediated atherosclerosis^18^, and neurodegenerative disease^13,19–21^ and is deregulated in cancer^22^. Understanding how TFEB is regulated in response to nutrient limitation is therefore a key issue.

Here we found that TFEB has a regulated nuclear export signal (NES) in which phosphorylation at the ERK/mTORC1 phosphorylation site at S142 primed for phosphorylation by GSK3β at S138. Phosphorylation at both sites was required for efficient nuclear export and GSK3β was inhibited via AKT downstream from mTORC2 in response to glucose limitation. Consequently, TFEB nuclear export was inhibited by limitation of either amino acids or glucose. The results establish that nuclear export is a critical nexus for regulation of TFEB subcellular localization.

## Results

### TFEB contains a nuclear export signal

**Fig. 1:**
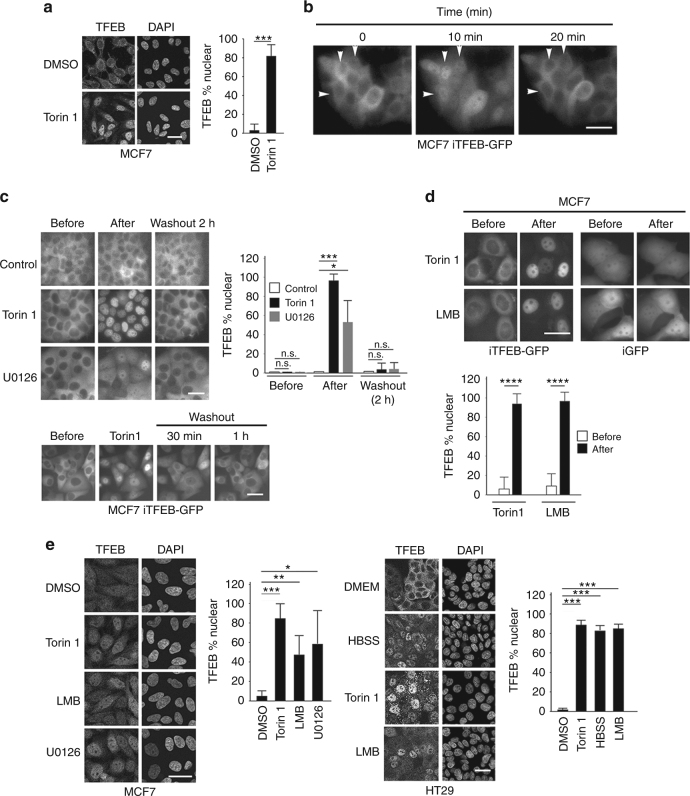
TFEB is subject to nuclear export. **a** Immunofluorescence with indicated antibodies using control MCF7 cells or those treated with Torin 1 (250 nM, 1 h). *n* > 30 cells per condition. **b** Real time imaging of MCF7 stable cell lines expressing doxycycline-inducible TFEB-GFP (iTFEB-GFP) imaged at indicated intervals in the absence of doxycycline. Images derived from Supplementary Movie 1. **c** Live cell imaging of MCF7 iTFEB-GFP cells before and after 1 h Torin 1 (250 nM) or 2 h U0126 (20 μM) treatment or after removal of the drug (upper panels) as indicated, or 30′ and 1 h after removal of Torin 1 (lower panels). *n* > 30 cells per condition. **d** Imaging of MCF7 iTFEB-GFP or iGFP cells before and after LMB (2 h; 20 nM) treatment. Quantification is derived from one experiment that is representative of 2 independent experiments. *n* > 30 cells per condition. **e** Immunofluorescence using MCF7 or HT29 cells and indicated antibodies with or without treatment with Torin 1 (1 h; 250 nM), LMB (2 h; 20 nM), and U0126 (3 h; 20 μM). *n* > 500 cells per condition. Details of all quantifications including number of replicates and cells imaged are provided in Supplementary Table 1 and Methods. Scale bars = 20 μM. Error bars = SD. Student’s *t*-test; *P < 0.05, **P < 0.01, ***P < 0.001, ****P < 0.0001, n.s. not significant

Under standard culture conditions endogenous TFEB was localized to the cytoplasm in the breast cancer cell line MCF7, but was relocated to the nucleus on addition of the mTOR inhibitor Torin 1 (Fig. 1a), indicating that in these cells mTOR controls TFEB localization. As most studies examine the steady state location of TFEB, we established a stably expressed GFP-reporter system in which the dynamics of TFEB cytoplasmic-nuclear shuttling could be examined in real-time by using MCF7 cells in which TFEB-GFP was under the control of a doxycycline-inducible promoter. In this cell line, in the absence of doxycycline, the cytoplasmic localization of the low basal level of TFEB-GFP reflected that of the endogenous protein. Examination of TFEB-GFP under these conditions revealed that TFEB subcellular localization was highly dynamic; over the course of 20 min TFEB in some cells was seen to accumulate in the nucleus and then return to the cytoplasm (Fig. 1b; Supplementary Movie 1), presumably indicating that TFEB responds to changing intracellular nutrient availability even within cells grown in a nutrient rich environment.

The dynamic switching between nuclear and cytoplasmic TFEB observed in some cells over time raised the possibility that in addition to cytoplasmic retention, TFEB could be subject to nuclear export. We therefore induced TFEB-GFP nuclear accumulation with the mTOR inhibitor Torin 1 and observed the location of TFEB over time following the removal of the drug. The results (Fig. 1c) revealed that TFEB was largely re-localized to the cytoplasm within 2 h (upper panels) or 30 min (lower panels). The rapid accumulation of TFEB in the cytoplasm would be consistent with its active nuclear export. Similar results were observed following addition and subsequent removal of the MEK inhibitor U0126 that can also trigger TFEB nuclear accumulation by preventing ERK-mediated phosphorylation on S142^11^. In agreement with previous observations^1^, U0126 was less efficient than Torin 1 at inducing nuclear TFEB since it does not affect cytoplasmic retention mediated by mTORC1 phosphorylation at S211^11^.

The presence of an NES in TFEB was confirmed using leptomycin B (LMB), a drug that prevents nuclear export by blocking the interaction between CRM1 and export signals^23^. Whereas LMB induced nuclear accumulation of TFEB-GFP in a similar fashion to Torin 1 (Fig. 1d), strongly suggesting that TFEB has an NES, it had no effect on the intracellular distribution of GFP stably expressed in MCF7 cells. Using Torin 1 and U0126 treatment as positive controls, nuclear accumulation of endogenous TFEB was also observed in MCF7 cells in response to LMB (Fig. 1e). Similar results were also obtained in the colon cancer cell line HT29 using Torin 1, and medium lacking amino acids but containing glucose (HBSS) as positive controls.

**Fig. 2:**
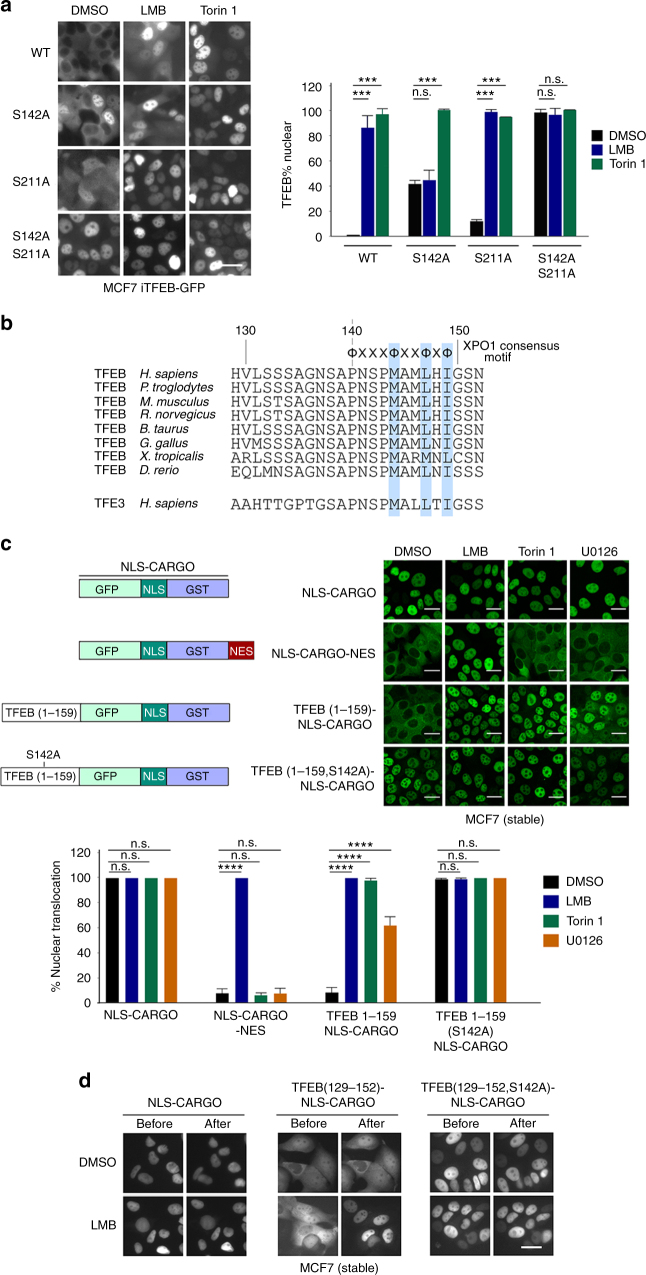
Identification of a TFEB nuclear export signal. **a** Live cell fluorescence images of indicated WT and mutant TFEB-GFP proteins in stably expressing MCF7 cells. Cells were treated with DMSO, LMB (20 nM; 2 h) or Torin 1 (20 nM; 1 h). *n* > 30 cells per condition. **b** Alignment of TFEB amino acids 129–152 and equivalent TFE3 residues. Amino acids highlighted in blue conform to the consensus for an NES. **c** Live cell fluorescence imaging of MCF7 cells stable expressing indicated fusion proteins imaged before or after treatment with LMB (2 h; 20 nM), Torin 1 (1 h; 250 nM), or U0126 (3 h; 20 μM). Imaging was performed after fixation. *n* > 50 cells per condition. Error bars = SD. Student’s *t*-test; ***P < 0.001, ****P < 0.0001, n.s. not significant. **d** Real time fluorescence imaging of MCF7 cells stably expressing the indicated fluorescent TFEB (129–152) cargo vectors before or after treatment with LMB (20 nM; 2 h). Scale bars = 20 μM

To examine the potential relationship between the putative NES and TFEB phosphorylation we studied the effect of LMB on TFEB nuclear accumulation in cell lines engineered to express stably low levels of WT and phosphorylation site mutated TFEB-GFP. In agreement with previous work^6,11^, while WT TFEB is largely cytoplasmic, mutation of S142 or S211 alone led to an increase in TFEB nuclear localization (Fig. 2a), though the effect of mutation of S211 was less pronounced than mutation of S142 as previously observed^1^. As expected, mutation of both S142 and S211 to alanine abolished cytoplasmic retention of TFEB. These observations are consistent with previous work describing the role of S142 and S211 in regulating TFEB nuclear/cytoplasmic distribution^1,6,11,12^. Using Torin 1 as a positive control for TFEB nuclear translocation, LMB dramatically increased the nuclear localization of the WT protein, but the proportion of nuclear S142A mutant was largely unaffected by LMB treatment. By contrast, the nuclear localization of the S211A mutant was strongly enhanced by LMB treatment indicating that export was independent of S211 modification. Collectively these data are consistent with phosphorylation of S142, but not S211, being important for nuclear export, while S211 is important for cytoplasmic retention of TFEB as previously described^6^.

Examination of the TFEB amino acid sequence in the vicinity of S142 led us to identify a consensus hydrophobic NES (Fig. 2b) that was highly evolutionarily conserved in the TFEB family as well as in the related factor TFE3. To test whether this region of TFEB was indeed an NES, we designed a cargo vector in which GFP is expressed as a chimera with GST to increase its size so as to prevent passive diffusion to the nucleus, together with a canonical PKKKRKV nuclear import signal from the SV40 large T-antigen^24^. This chimeric protein is constitutively nuclear and is unaffected by treatment of cells with LMB, Torin 1 or U0126 (Fig. 2c). The GFP-NLS-GST cargo can be relocalized to the cytoplasm by addition of a classical LALKLAGLDI NES from the cAMP-dependent protein kinase inhibitor (PKI)^25^. In this case addition of LMB prevents nuclear export and the cargo is relocalized to the nucleus, but this NES is unresponsive to Torin 1 or U0126. By contrast, substitution of the PKI NES with amino acids 1–159 from TFEB (to exclude any influence of cytoplasmic retention by S211 phosphorylation) led to cytoplasmic accumulation in the absence of drug treatment, but LMB as well as Torin 1 effectively drove its nuclear accumulation. In this reporter U0126 also induced increased nuclear accumulation of TFEB, though less efficiently than Torin 1, and mutation of S142 to alanine led to nuclear accumulation that was unaffected by any drug treatment. To verify that the region containing S142 and the hydrophobic residues were sufficient to mediate nuclear export we inserted TFEB sequences 129–152 into the cargo vector and followed its localization in real time before and after LMB treatment. The result (Fig. 2d) revealed that residues 129–152 was accumulated in the nucleus within 1 h of LMB treatment. Moreover, mutation of S142 to alanine led to constitutive nuclear localization of the 129–152 cargo reporter. Collectively, these data suggest that TFEB has a NES that is dependent on S142 phosphorylation.

**Fig. 3:**
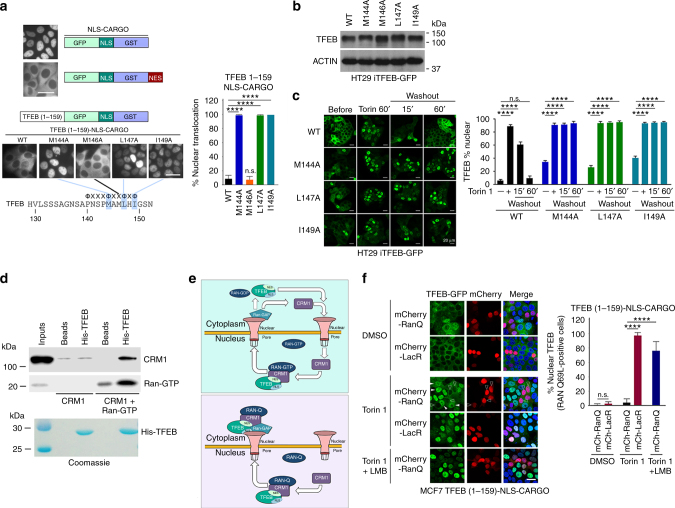
TFEB is subject to CRM1-mediated nuclear export. **a** Live cell steady state images of indicated cargo vectors stably expressed in MCF7 cells. *n* > 120 cells per condition. **b** Western blot of HT29 cells expressing doxycycline-inducible WT or indicated mutant TFEB-GFP (−Dox). **c** Fluorescence images of HT29 cells stably expressing indicated WT and mutant TFEB-GFP treated with Torin 1 (25 nM) for 1 h before cells were washed and placed in Torin 1-free medium for 15 or 60 min. *n* > 300 cells per condition. **d** Western blot using indicated antibodies to detect CRM1 and RAN after pull down of immobilized HIS-tagged TFEB (aas 1–200). All proteins were bacterially expressed and purified. Purified TFEB was visualized by Coomassie staining (lower panel). **e** Schematic depicting the role of CRM1 and RAN in nuclear export using TFEB as a potential substrate. Top: RAN-WT; Bottom, RAN-Q69L. See text for details. **f** Fluorescence images of steady state subcellular localization of MCF7 cells stably expressing the TFEB 1-159-NLS-cargo together with either mCherry-LacR or mCherry RAN-Q69L. Cells were treated with DMSO, Torin 1 (250 nM) alone or with LMB (20 nM) as indicated. Subcellular localization of TFEB-GFP was quantified for cells expressing mCherry. *n* > 100 cells per condition. In the RanQ experiment, only mCherry positive cells were counted. Scale bars = 20 μM. Error bars = SD. Student’s *t*-test; ****P < 0.0001, n.s. not significant

To verify the role of the hydrophobic residues in the consensus TFEB NES identified, we mutated M144, M146, L147, and I149 to alanine and tested their effect on the steady state localization of stably expressed 1–159 cargo reporter (Fig. 3a). The results revealed loss of cytoplasmic localization with the M144, L147, and I149 to alanine mutants indicating that they contribute to the function of the TFEB NES. By contrast mutation of M146 to alanine had no effect on TFEB subcellular localization and therefore provided a negative control for the effect of mutation of the other hydrophobic mutants.

These observations were confirmed using stable cell lines expressing similar levels of full length WT and mutant TFEB-GFP (Fig. 3b) which, in contrast to the cargo vectors, contain S211 that mediates cytoplasmic retention through interaction with 14-3-3 proteins. WT and mutant proteins were induced to translocate to the nucleus using Torin 1 and their capacity to be re-exported on Torin 1 washout examined over time. As with the cargo vectors, mutation of M144, L147 or I149 prevented the nuclear export observed using the WT protein (Fig. 3c).

The ability of LMB to prevent TFEB nuclear export implies that as it transits the nuclear pore the TFEB NES is transiently bound by the exportin CRM1 in complex with RAS-related GTPase RAN. The ability of CRM1 to bind TFEB in vitro was initially verified using bacterially expressed and purified proteins in the presence of a twofold excess of RAN-GTP (Fig. 3d). The release of export cargos from CRM1 requires hydrolysis of GTP to GDP by RAN that then allows CRM1 and any cargo with a nuclear import signal such as TFEB, to be recycled to the nucleus (Fig. 3e, upper panel). GTP hydrolysis by RAN can be prevented by a Q69L mutation, and as this mutant retains the ability to form the ternary CRM1-RAN-cargo complex, the expression of RAN-Q69L permits nuclear export, but prevents dissociation of CRM1 from the cargo (Fig. 3e, lower panel). If TFEB interacts with CRM1 in vivo as expected, expression of a RAN-Q69L mutant should trap TFEB in the cytoplasm by preventing dissociation from the CRM1-RAN complex after export. To test this, we expressed the RAN-Q69L mutant as a fusion with mCherry in MCF7 cells expressing TFEB-GFP, using mCherry-LacR^26^ as a negative control. Under steady state conditions when TFEB is retained in the cytoplasm the expression of mCherry-RAN-Q69L or mCherry-LacR made little difference to the localization of TFEB (Fig. 3f). Nuclear accumulation of TFEB was also observed in cells treated with Torin 1 irrespective of expression of the control mCherry-LacR. By contrast, mCherry-RAN-Q69L expression prevented nuclear accumulation of TFEB in the presence of Torin 1, an effect reversed by LMB. The effect of LMB indicates that TFEB cytoplasmic accumulation in the presence of Torin 1 and RAN-Q69L expression requires prior nuclear export. These data are consistent with a model (Fig. 3e, lower panel) in which following its nuclear import, for example in response to amino acid starvation or Torin 1 treatment, TFEB is then subject to CRM1 and RAN-dependent export. Blocking the dissociation of the TFEB-CRM1-RAN ternary complex in the cytoplasm after export using RAN-Q69L then prevents TFEB nuclear import and leads to the observed cytoplasmic accumulation.

### TFEB S138 phosphorylation by GSK3β is primed by S142 phosphorylation

**Fig. 4:**
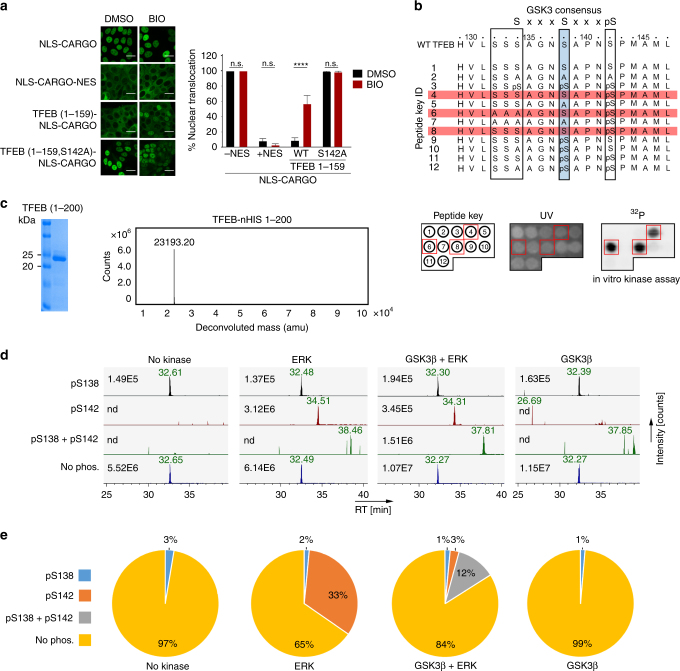
Phosphorylation at S142 primes for GSK3β phosphorylation at S138. **a** Fluorescence images of cells expressing indicated WT and mutant cargo vectors in the presence and absence of BIO (10 μM). *n* > 50 cells per condition. Error bars = SD. Scale bar = 20 μM. ****p < 0.0001, n.s. not significant. **b** TFEB amino acids 129–152 showing the GSK3β consensus sequence. The S138 GSK3β site is highlighted in blue, the ERK/mTOR phosphorylation site S142 is boxed, as are three additional potential GSK3β sites that could be primed by phosphorylation at S138. Peptides 1–12 were spotted onto a membrane (see key corresponding to peptides in lower left panel) and subject to GSK3β phosphorylation in vitro. Peptides highlighted in red were phosphorylated by GSK3β. Lower panels: Peptide key corresponding to peptides above spotted onto a membrane (left) and visualized by UV (middle). The result of the GSK3β kinase assay is shown in the right panel and the red boxes correspond to phosphorylated peptides highlighted in red above. **c** Coomassie-stained gel of bacterially expressed and purified 6xHIS-tagged TFEB amino acids 1–200 (left) and associated Mass Spec spectrum (right). **d** Extracted ion chromatograms of tryptic peptides covering S138 and S142 in their differential phosphorylation states after indicated kinase treatment. A peptide with a single phosphorylation on S138 was detected at low intensity in all samples, including those not treated with GSK3β, while a peptide with a phosphorylation event on S142 was only detected in the ERK and GSKβ + ERK treated samples. Peptides dually phosphorylated on both S138 and on S142 were detected only after GSKβ + ERK treatment. Numbers represent maximum ion count for each peptide/phospho-peptide. **e** Quantification of TFEB in vitro kinase/Mass Spec data. The pie-charts illustrate the signal contribution of each (phospho-)peptide variant to total signal intensity covering the analyzed phosphorylation locus after kinase treatments. Assuming that each peptide variant has identical ionization characteristics, the charts reflect the stoichiometry of the different phospho-proteoforms of TFEB after kinase treatment

The results so far suggest that TFEB has an NES located in proximity to S142, an mTOR and ERK phosphorylation target that regulates nuclear localization. S142 lies close to the recently identified GSK3β phosphorylation site at S138^13^ that has also been implicated in promoting cytoplasmic TFEB localization independently of mTOR. How S138 impacts TFEB subcellular localization is unclear. We therefore used BIO, a selective GSK3β inhibitor to determine the effect on the subcellular location of the TFEB 1–159 cargo vector. While BIO had no effect on the location of the control NLS and NES-containing reporters, it promoted a substantial increase in nuclear accumulation of the TFEB 1–159 reporter (Fig. 4a), raising the possibility that GSK3β phosphorylation at S138, like phosphorylation at S142, could contribute to TFEB nuclear export. However, although GSK3β frequently uses a priming phosphorylation event to efficiently trigger phosphorylation at its consensus recognition motifs, previous work^13^ reported that GSKβ-mediated phosphorylation of S138 and its effect on TFEB subcellular localization is mTOR independent. Nevertheless, the GSK3β site at S138 could potentially be primed by mTOR or ERK-mediated phosphorylation on S142 (Fig. 4b). Moreover, since the previous study^13^ used bacterially expressed TFEB as a substrate for their in vitro GSK3β kinases assays, the potential of a phosphorylation event at S142 priming GSK3β-mediated phosphorylation at S138 was not assessed. We therefore made use of peptide arrays in which the key residues spanning TFEB 129–152 were unmodified, mutated to alanine, or phosphorylated (Fig. 4b). We also examined the possibility of phosphorylation at the additional potential GSKβ sites at S132, S133 and S134. The results revealed no detectable phosphorylation above background of the WT peptide (Fig. 4c). By contrast efficient phosphorylation was observed if the mTOR/ERK phosphorylation site S142 was phosphorylated and S138 intact. Mutation of S138 to alanine completely blocked phosphorylation stimulated by phospho-S142. In this assay, no phosphorylation was detected on S132–134, even if S138 and/or S142 were phosphorylated. We also employed a complementary mass spectrometry approach in which the N-terminal region of TFEB (residues 1–200) was bacterially expressed, purified and phosphorylated in vitro using ERK or GSK3β alone or in combination (Fig. 4c–e). Quantification of the phosphorylation events (Fig. 4d, e) confirmed that ERK could phosphorylate S142, but not S138, whereas S138 was not phosphorylated by GSK3β above background unless S142 was also phosphorylated by ERK. These data confirmed the peptide kinase assays and again suggested that phosphorylation of S138 is dependent on prior phosphorylation of S142, a conclusion in agreement with previous high-throughput phosphoproteomic analyses^27,28^ in which TFEB peptides phosphorylated at S142 but not S138 were detected, but those phosphorylated at S138 were also phosphorylated at S142.

**Fig. 5:**
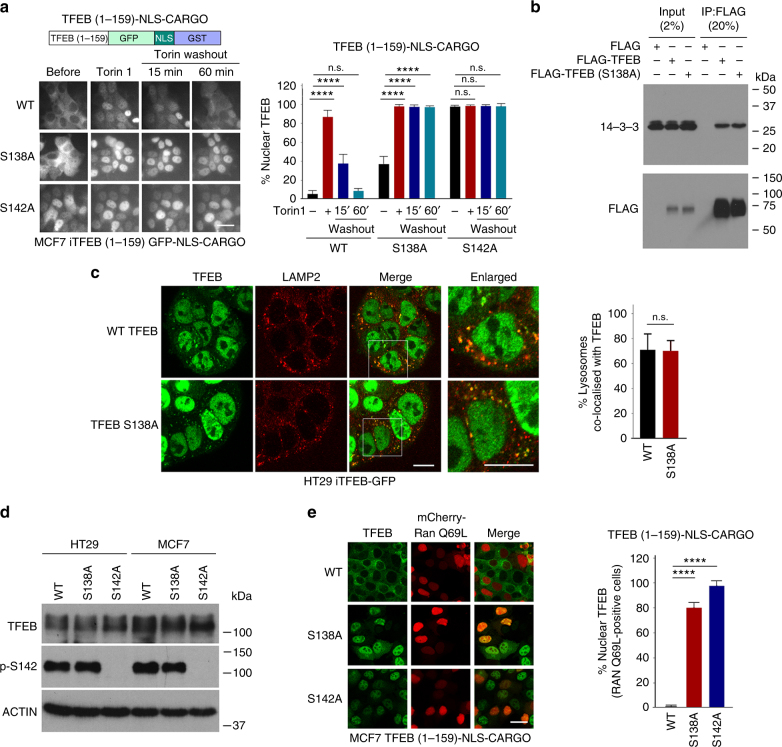
S138 and S142 control nuclear export. **a** Live cell imaging of MCF7 cell lines stably expressing the indicated TFEB-cargo vectors. Cells were treated with Torin 1 (20 nM; 1 h) before the medium was changed and cells imaged at indicated times. *n* > 200 cells per condition. **b** Immunoprecipitation assay of extracts of cells transfected with FLAG-TFEB WT or S138A mutant. After immunoprecipitation, western blots were probed with anti-FLAG and anti-14-3-3 antibody. **c** Immunofluorescence of HT29 cells expressing stable doxycycline-inducible TFEB WT or S138A mutant treated with Torin 1 (1 h; 250 nM) showing co-localization with lysosomes marked using anti-LAMP2. Scale bar 10 μM. For lysosome co-localization quantification, lysosomes were stained by anti-LAMP2 antibody and their total numbers were counted. The percentage of TFEB lysosome co-localization was defined by the percentage of lysosomes with positive TFEB-GFP co-localization. *N* > 50 cells per condition. **d** Western blot of HT29 or MCF7 cells stably expressing indicated WT or mutant TFEB probed with anti-TFEB antibody or an antibody recognizing specifically phospho-S142. Actin is a loading control. **e** Fluorescence assays for MCF7 cells stably expressing doxycycline-inducible WT TFEB-GFP and S138A and S142A mutants in the absence of doxycycline transfected with mCherry RAN-Q69L. Quantification is for mCherry-RAN Q69L-expressing cells. *n* > 40 cells per condition. Student’s *t*-test; *P < 0.05, **P < 0.01, ***P *<* 0.001, ****P *<* 0.0001. Error bars = SD. Scale bars = 20 μM

To test the effect of S138 and S142 mutations on nuclear export, WT and alanine substitutions were introduced in the context of the TFEB 1–159 cargo vector stably expressed in MCF7 cells. This enables the impact of these mutations to be assessed in live cells in the absence of cytoplasmic retention by phosphorylated S211. In untreated cells the WT protein was largely cytoplasmic, whereas the S142A mutant exhibited an increased nuclear localisation (Fig. 5a). Close to 40% of cells expressing the S138A mutant also exhibited a strong nuclear localization. In response to Torin 1, all proteins were localized to the nucleus. On Torin 1 washout, in most cells the WT protein returned to the cytoplasm within 60 min, but both the S138A and S142A mutants were retained in the nucleus consistent with phosphorylation at both residues being required for efficient nuclear export.

Previous observations^13^ showed that an S138A mutation reduced binding between TFEB and the 14-3-3 protein that binds phosphorylated S211 and abolished also co-localization with lysosomes. However, we found instead by co-immunoprecipitation that the S138A mutant did not affect interaction with 14-3-3 proteins (Fig. 5b). Nor did the S138A mutation affect co-localization with lysosomes (Fig. 5c) under conditions where TFEB recruitment to lysosomes is stimulated by mTOR inhibition as described previously^1,29^. Importantly, the S138A mutation failed to prevent phosphorylation of TFEB on S142 using an antibody specifically recognizing this site (Fig. 5d). In addition, ectopic expression of RAN-Q69L failed to induce cytoplasmic accumulation of the TFEB S142A and S138A mutants (Fig. 5e), consistent with them failing to undergo efficient nuclear export. Together these results suggest that mTOR or ERK phosphorylation on S142 stimulates GSK3β phosphorylation at S138 and that the dual phosphorylation event is necessary for nuclear export.

### TFEB nuclear export is regulated by glucose

**Fig. 6:**
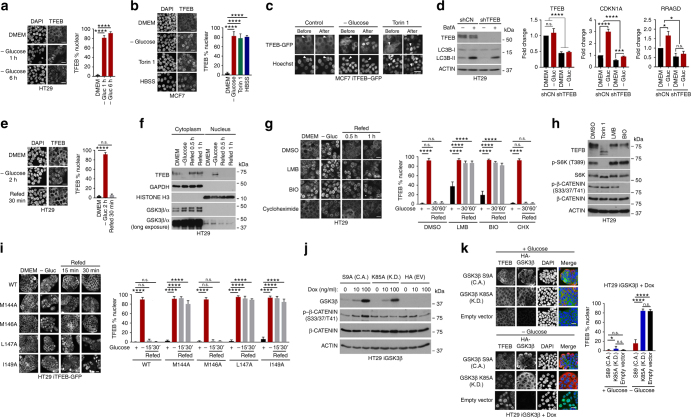
TFEB nuclear export is regulated by Glucose. **a** Immunofluorescence of HT29 cells grown under indicated conditions. *n* > 100 cells per condition. **b** Immunofluorescence of MCF7 cells grown in DMEM, minus glucose, or +Torin 1 (250 nM) or HBSS. *n* > 30 cells per condition. **c** Live cell imaging of MCF7 iTFEB-GFP cells before or after glucose deprivation or Torin 1 (250 nM) treatment. **d** Left: western blot of HT29 cells expressing control shRNA or shTFEB grown in the presence or absence of Bafilomycin A (10 μM). Right: real time qRT PCR of CDKN1A (p21) and RRAGD mRNAs from shControl or shTFEB cells grown with or without glucose (3 h) with or without shTFEB. *n* = 3 individual experiments. Error bars = SD. Two-way ANOVA, *P *<* 0.05, ***P *<* 0.001, ****P *<* 0.0001, n.s. not significant. **e** Immunofluorescence of HT29 cells grown under indicated conditions. *n* > 100 cells per condition. **f** Western blot of fractionated HT29 cells grown under indicated conditions. **g** Immunofluorescence of HT29 cells grown in DMEM, or starved of glucose 1 h before refeeding with glucose alone or with LMB (20 nM), BIO (10 μM) or cycloheximide (50 μg ml^−1^). *n* > 500 cells per condition. **h** Western blot of HT29 cells treated with Toirn 1 (250 nM), LMB (2 nM) or BIO (10 μM) as indicated. **i** Fluorescence assay of TFEB-GFP WT and indicated mutants stable expressed in HT29 cells. Cells were grown in DMEM, starved of glucose for 2 h, then refed with glucose for 15 or 30 min and fixed before imaging. **j** Western blot of HT29 cells stably expressing doxycycline-inducible kinase dead (K.D.) or constitutively active (C.A.) HA-GSK3β and induced with 0, 10 and 100 ng ml^−1^ doxycycline for 20 h. **k** Immunofluorescence using anti-TFEB or anti-HA antibodies of HT29 cells expressing doxycycline-inducible constitutively active (C.A) or kinase dead (K.D.) GSK3β or empty vector grown in the presence of doxycycline (100 ng ml^−1^; 20 h) in the presence of glucose or deprived of glucose for 1 h. *n* > 200 cells per condition. Scale bars = 20 μM. Error bars = SD. Student’s *t*-test; *P < 0.05, **P *<* 0.01, ***P < 0.001, ****P *<* 0.0001

The previous identification of GSK3β as a regulator of TFEB activity used 5*β*-*O*-angelate-20-deoxyingenol (Hep14) to inactivate GSK3β via its ability to up-regulate Protein kinase C^13^. However, a physiological regulator of GSK3β-mediated phosphorylation of TFEB was not identified. We reasoned that while the mTORC1 complex would regulate TFEB subcellular localization in response to amino acid availability, a GSK3β and mTOR/ERK dual-dependent NES had the potential to integrate multiple nutritional inputs. The ability of TFEB to control expression of genes implicated in lipid metabolism^3^ provided an indication that TFEB activity should be responsive to not only amino acids, but also to carbon sources such as glucose. Indeed, it has recently been shown that mTORC1 can be inhibited by hexokinase in response to low glucose^30^. We therefore asked whether TFEB’s subcellular localization could be regulated by glucose availability. Using HT29 cells endogenous TFEB translocated to the nucleus on glucose deprivation (Fig. 6a). Similar results were observed using endogenous TFEB in MCF7 cells, using Torin 1 or HBSS as controls (Fig. 6b), or by using real-time imaging of the TFEB-GFP reporter (Fig. 6c).

To assess the impact of glucose deprivation on the ability of TFEB to regulate transcription, we performed quantitative real time PCR (qRT PCR) of mRNA derived from HT29 cells expressing a TFEB-specific shRNA or control shRNA grown in the presence or absence of glucose. Western blotting of the shTFEB cells revealed that TFEB was efficiently silenced (Fig. 6d, left panel) and that silencing of TFEB reduced expression of the autophagy marker LC3B-II in both the presence and absence of Bafilomycin A, an inhibitor of autophagosome-lysosome fusion. To examine the consequence of TFEB nuclear accumulation in response to glucose limitation, we chose to examine the expression of p21 (*CDKN1A*) a well-characterized target of the TFEB-related factor MITF^31^, and RAS-related GTP-binding protein D (*RRAGD*)^32^. In cells expressing control shRNA both targets were up-regulated following glucose deprivation (Fig. 6d, right panel). By contrast, cells expressing shTFEB failed to induce significantly either CDKN1A or RRAGD mRNA expression in response to low glucose. Thus, TFEB activates target genes in response to glucose limitation.

Glucose deprivation followed by re-feeding led to relocalization of TFEB to the cytoplasm within 30 min (Fig. 6e), a result confirmed using cell fractionation (Fig. 6f) which also revealed a fraction of GSK3β in the nucleus as previously described^33^. Both LMB and the GSK3β inhibitor BIO inhibited the re-localization of TFEB to the cytoplasm on re-addition of glucose following glucose deprivation (Fig. 6g). Since both inhibitors did not decrease phosphorylation of the mTORC1 target S6K (Fig. 6h), their ability to promote nuclear localization of TFEB is independent of the mTORC1 inhibition observed on amino acid limitation. The accumulation of TFEB in the cytoplasm was not inhibited by the protein synthesis inhibitor cycloheximide, indicating that the rapid cytoplasmic accumulation observed upon re-feeding with glucose did not arise from de novo protein synthesis. In HT29 cells engineered to stably express doxycycline-inducible TFEB-GFP or TFEB NES mutants, WT TFEB entered the nucleus in response to glucose deprivation and returned to the cytoplasm on glucose re-feeding (Fig. 6i). However, export triggered by glucose refeeding was prevented using the M144A, L147A, and I149A mutants. The M146A mutant served as a control and behaved like the WT protein. These results are consistent with GSK3β-activated TFEB export being glucose responsive.

To confirm the role of GSK3β in TFEB nuclear export following glucose re-feeding we generated stable HT29 cell lines expressing doxycycline-inducible constitutively active (C.A.) GSK3β, bearing an alanine mutation in the inactivating Serine 9 phosphorylation site (S9A), or a kinase dead (K.D.) mutant, containing an alanine substitution in lysine 85 that is required to coordinate ATP in the catalytic site. The relative expression of the two mutants in response to doxycycline is shown in Fig. 6j, where the C.A. mutant, but not the K.D. mutant, can induce phosphorylation of β−catenin, a well-characterized GSK3β target. In the presence of doxycycline and glucose endogenous TFEB was localized to the cytoplasm irrespective of which GSK3β mutant was expressed (Fig. 6k). By contrast, in the absence of glucose the constitutively active GSK3β mutant largely prevented the nuclear accumulation of TFEB that was observed in control cells or those expressing the kinase dead mutant. These data are consistent with GSK3β inactivation in response to low glucose being required to prevent efficient TFEB nuclear export.

### Low glucose activates an mTORC2-AKT-GSK3β axis

**Fig. 7:**
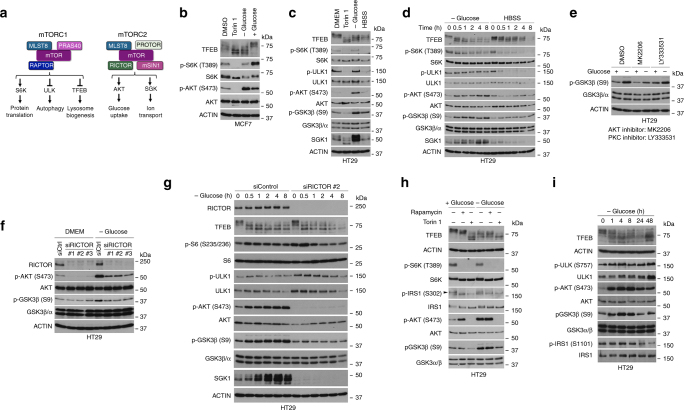
mTORC2 regulates GSK3β in response to glucose limitation. **a** Schematic showing the mTORC1 and mTORC2 complexes and downstream targets. **b**–**d** Western blots using indicated antibodies of HT29 cells grown under indicated conditions. Unless otherwise indicated cells were placed in HBSS for 2 h, or treated with Torin 1 (250 nM) or starved of glucose for 2 h and where indicted refed glucose for 1 h. **e** Western blot using indicated antibodies of HT29 cells grown in DMEM or starved of glucose for 4 h and treated with DMSO, MK2206 (3 μM), or LY333531 (5 μM). **f** Western blot using indicated antibodies of HT29 cells. 48 h after transfection with RICTOR-specific or control siRNAs cells were glucose starved for 2 h, as indicated. **g** Western blot using indicated antibodies of HT29 cells transfected with control or RICTOR-specific siRNA for 48 h prior to glucose starvation for indicated times. **h** Western blot using indicated antibodies of HT29 cells grown in the presence of absence of glucose (4 h) of cells treated with Rapamycin (1 μM) or Torin 1 (250 nM). **i** Western blot using indicated antibodies of HT29 cells following glucose starvation for indicated times

Mammalian cells possess two major mTOR-containing complexes, mTORC1 and mTORC2 (Fig. 7a). Since mTORC1 has been reported to control TFEB subcellular localization we anticipated glucose deprivation, like amino acid starvation, would inhibit mTORC1 activity. As expected, in MCF7 cells Torin 1 decreased phosphorylation of the mTORC1 target S6K, as well as the mTORC2 target S473 AKT (Fig. 7b), and also generated a faster migrating form of TFEB characteristic of its de-phosphorylation and consequent nuclear accumulation^34^. Surprisingly however, glucose limitation, that can trigger nuclear accumulation of TFEB, did not decrease S6K phosphorylation, suggesting that mTORC1 activity was largely unaffected. Instead we observed increased phosphorylation of AKT S473, an mTORC2 target. Glucose limitation, like Torin 1 also increased the migration of TFEB, consistent with nuclear entry under these conditions being triggered by activation of TFEB phosphatases as has been observed in response to amino acid starvation^34^. Re-feeding with glucose reversed the effect on TFEB migration and similarly decreased the levels of pS473 AKT seen under glucose deprivation. Glucose addition also increased pS6K levels, consistent with addition of glucose stimulating mTORC1 signaling, as has been observed previously^35,36^. Glucose-mediated activation of mTORC1 would facilitate cytoplasmic retention of TFEB. Similar results were obtained in HT29 cells (Fig. 7c) where phosphorylation of the mTORC1 targets S6K and ULK1 were both decreased by Torin 1 or HBSS, but not by glucose limitation. Instead glucose starvation triggered increased pS473 AKT and elevated SGK expression, hallmarks of mTORC2 activation. Moreover, glucose limitation also led to increased modification of GSK3β on S9, an inactivating phosphorylation event mediated by activated AKT. By contrast pS9 GSK3β was slightly decreased by Torin 1, consistent with reduced AKT phosphorylation, and was only marginally increased by HBSS. A time course (Fig. 7d) confirmed that HBSS substantially decreased phosphorylation of the mTORC1 targets S6K and ULK1. By contrast, glucose limitation did not significantly affect ULK1 phosphorylation, and pS6K was only moderately reduced at later time points. Importantly, glucose deprivation, but not HBSS, increased phosphorylation of AKT and its target GSK3β and also induced SGK expression. Induction of pS473 AKT and pS9 GSK3β occurred within 30 min, of glucose depletion, consistent with the observed rapid accumulation of TFEB in the nucleus. MK2206, an AKT inhibitor, but not the PKC inhibitor LY333531, prevented phosphorylation of S9 GSK3β in response to glucose deprivation (Fig. 7e) and confirmed that glucose starvation inactivated GSK3β via AKT.

One of the key regulators of AKT S473 phosphorylation is the mTORC2 complex that was recently identified as being activated by glutamine catabolite levels and glucose limitation^37^. Previous work showed that siRNA-mediated depletion of RICTOR, a key mTORC2-specific subunit, failed to affect TFEB or TFE3 subcellular localization^19,29,38^. However, those experiments were not performed under the glucose starvation conditions that inactivate GSK3β to block nuclear export. We therefore used three different siRNAs to deplete RICTOR and subsequently deprived cells of glucose. Western blotting revealed that depletion of RICTOR largely prevented glucose depletion-induced AKT S473 phosphorylation and also blunted the increase in GSK3β S9 phosphorylation (Fig. 7f). A time course of glucose starvation in control or RICTOR-depleted cells (Fig. 7g) confirmed that RICTOR depletion does not affect the increase in mobility of TFEB that occurs on glucose limitation, consistent with nuclear entry being triggered by activation of TFEB phosphatases as previously described^34^. However, RICTOR depletion severely blunted the effects of glucose limitation on phosphorylation of AKT and GSK3β as well as the accumulation of SGK.

AKT can be activated in response to rapamycin that inhibits mTORC1 but not mTORC2^39–41^. This occurs because of a negative feedback loop in which inhibition of mTORC1 and reduced S6K activity relieves inhibition of IRS1/2 that enhance receptor-mediated PI3K signaling to AKT^39–41^. In principle therefore, because AKT activation is expected to inhibit GSK3β and consequently inactivate the TFEB NES, rapamycin should promote nuclear accumulation of TFEB. Yet previous work has established that rapamycin does not trigger TFEB nuclear localization^1,6^, an observation potentially incompatible with our conclusion that AKT activation in response to glucose limitation inhibits the GSK3β-dependent TFEB NES. To investigate the possible involvement of the negative feedback loop in regulation of TFEB in response to glucose limitation we compared the response of cells to rapamycin, which inhibits mTORC1, to Torin 1 that inhibits both mTORC1 and mTORC2 in the presence and absence of glucose. The results (Fig. 7h) were striking. In the absence of inhibitors, as expected, glucose limitation activated AKT and downstream GSK3β phosphorylation but did not affect S6K phosphorylation. Both rapamycin and Torin 1 inhibited S6K phosphorylation irrespective of the presence of glucose. In the presence of glucose, rapamycin induced moderate AKT-phosphorylation, consistent with activation of the IRS-dependent feedback loop, but remarkably it did not lead to phosphorylation of S9 of GSK3β. By contrast, glucose limitation triggered phosphorylation of both AKT and GSK3β irrespective of the presence of rapamycin. Unlike rapamycin, Torin 1 inhibited AKT phosphorylation in both the presence and absence of glucose, and also prevented the glucose deprivation-induced phosphorylation of GSK3β. Together with the fact that mTORC1-dependent phosphorylation of TFEB is rapamycin insensitive^1,6^, the inability of rapamycin-induced AKT activation to promote the inhibitory S9 phosphorylation of GSK3β may explain why rapamycin does not induce nuclear accumulation of TFEB, and also suggests that the IRS-mediated feedback loop does not significantly contribute to regulation of GSK3β-dependent TFEB nuclear export.

This conclusion was further confirmed by examining the longer term response to glucose limitation over 48 h (Fig. 7i). While activation of AKT (pS473) and inhibition of GSK3β (pS9) occurs early, reduced phosphorylation of IRS1 on the S6K target S1101, a hallmark of activation of the IRS-mediated feedback loop, only occurs by 24 h following glucose limitation. Again, since TFEB enters the nucleus within 1 to 2 h following glucose starvation, it is unlikely the IRS feedback loop plays a significant role in regulating nuclear localization of TFEB in response to glucose limitation.

## Discussion

Over recent years TFEB has emerged as a central effector of the response to amino acid limitation. Under amino acid rich conditions, TFEB is phosphorylated by mTORC1 on S211 leading to its cytoplasmic retention. In response to amino acid limitation, mTORC1 is inactivated to prevent de novo phosphorylation of TFEB, while the activation of phosphatases such as calcineurin^34^ lead to its de-phosphorylation. As a consequence, dephosphorylated TFEB is released from its cytoplasmic anchor and enters the nucleus where it regulates genes implicated in lysosome biogenesis and autophagy as part of an adaptive response directed towards rebalancing nutrient supply with demand. Even in nutrient rich conditions the activity of mTORC1 (reflecting amino acid availability), RAG proteins^1,42,43^ or TFEB phosphatases, including calcineurin^34^, is likely to be dynamic. Any TFEB that is not stably sequestered in the cytoplasm will therefore undergo nuclear import mediated by its basic region. Thus, without the efficient NES identified here, even under nutrient–rich conditions, TFEB will over time accumulate in the nucleus as is observed when export is blocked using LMB. As such, the adaptive response to nutrient limitation afforded by the cytoplasmic sequestration of TFEB is critically dependent on efficient nuclear export; TFEB flux through the import-export cycle will provide a means to constantly adjust the balance of nutrient supply-demand by tuning the expression of its target genes. As such, failure to export TFEB, either via aberrant expression of importin 8 in pancreatic cancer^22^, or via deregulation of the events controlling nuclear export via S142 and S138 phosphorylation, may maintain cells in a catabolic state or at least diminish their metabolic flexibility in response to a changing microenvironment.

**Fig. 8:**
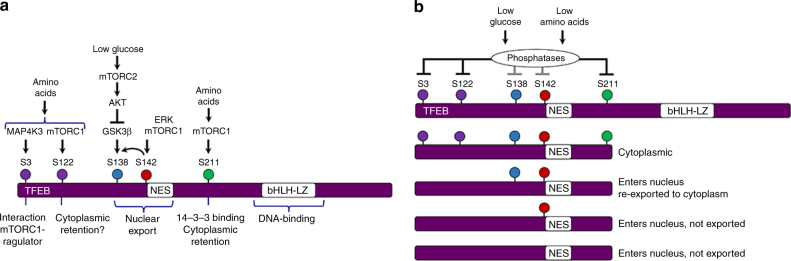
Model to explain regulation of TFEB subcellular localization. **a** Summary of key regulatory phosphorylation events and their impact on TFEB subcellular location. **b** Model to explain the potential regulation of TFEB when amino acids or glucose are limiting. Under these conditions increased phosphatase activity targeting S211, and likely other phosphorylation sites, will release TFEB from its cytoplasmic anchor. Whether TFEB is retained in the nucleus will be dependent on the phosphorylation status of S138 and S142. Absence of phosphorylation of S138 or S142 will lead to nuclear retention. Note however that in this model dephosphorylation of S142 or S138 is not a necessary pre-requisite for nuclear entry

Although the response of TFEB to amino acid starvation has been the focus of considerable attention, glucose limitation also promotes nuclear accumulation of TFEB. Unlike amino acid deprivation that triggers inactivation of mTORC1, in low glucose the rapid nuclear accumulation of TFEB is not associated with decreased mTORC1 signaling or activation of the IRS1 feedback loop. Nevertheless, both amino acid starvation and low glucose trigger a similar increase in TFEB mobility, strongly suggesting that nuclear accumulation is associated with activation of phosphatases that will reverse phosphorylation events that promote TFEB cytoplasmic retention. Of the phosphorylation sites known to make a substantial contribution to regulating TFEB subcellular localization, mechanistically only the role of phospho-S211 that mediates interaction with the 14-3-3 cytoplasmic anchor, and S3 that promotes binding to the mTORC-Ragulator complex have been well-defined (Fig. 8a). De-phosphorylation of S3, S211, and most likely S122^12^, will therefore enhance the probability of TFEB translocating to the nucleus (Fig. 8b), with additional residues in the C-terminal region also reported to be important under low serum conditions^5^. However, although S138 and S142 have also been identified as key regulators of TFEB subcellular localization, our observations implicating phosphorylation of these residues in nuclear export suggests there is no necessary pre-requisite for them to be dephosphorylated to promote TFEB nuclear entry. Indeed, a previous report^5^ has indicated that nuclear TFEB is at least partially phosphorylated. However, while nuclear entry as a consequence of de-phosphorylation of S3, S211, and S122 may be a critical first step in the adaptive response to nutrient limitation, for TFEB to function as a transcription factor its nuclear export must be prevented. In this respect, our observations on the role of S138 phosphorylation by GSK3β and its regulation in response to glucose limitation provide a key insight.

While many export signals are apparently not regulated, a few are controlled by phosphorylation, that of the yeast transcription factor Pho4 for example^44^. However, the NES in TFEB appears unique in that dual phosphorylation by distinct kinases provides an opportunity to control TFEB export through the integration of different inputs. Importantly, the phosphorylation events are linked. In contrast to a previous report that GSK3β phosphorylation of TFEB was independent of S142^13^, we show that in fact phosphorylation of S142, a target of both mTOR and ERK, dramatically enhances efficient phosphorylation of S138 by GSK3β, with dual phosphorylation being required for efficient nuclear export. These results provide a mechanistic explanation for previous observations^13,15,45^ suggesting inhibition of GSK3β can lead to nuclear accumulation of TFEB. However, although S138 and S142 are located adjacent to the NES and clearly are implicated in TFEB export, whether they do so by regulating access of CRM1 to the hydrophobic export signal, or possibly also by affecting retention of TFEB to a nuclear anchor, remains to be determined. Nevertheless, collectively our results are consistent with a model (Fig. 8) in which de-phosphorylation of TFEB plays a key role in promoting its nuclear entry in response to both amino acid or glucose starvation. Which residues are dephosphorylated to promote nuclear entry is currently unclear, and while GSK3β is inhibited by glucose limitation, we have not formally shown that low glucose decreases S138 phosphorylation. Nevertheless, in contrast to amino acid starvation, low glucose initially triggers nuclear entry of TFEB without substantially affecting mTORC1 signaling, while mTORC2, and not the IRS feedback loop, inactivates GSK3β and inhibits TFEB nuclear export. Our results suggest that in response to glucose levels mTORC1 and mTORC2 may therefore have complementary roles in regulating TFEB subcellular localization, with low glucose via mTORC2 inactivating GSK3β and the TFEB NES, but restoration of glucose stimulating mTORC1 signaling to promote cytoplasmic retention.

Under glucose limiting conditions, inactivation of the NES and TFEB nuclear retention is achieved via mTORC2 that triggers AKT-mediated inactivation of GSK3β in response to low glucose. Although AKT can be activated via the IRS1 feedback loop, we find no evidence for this feedback loop operating to regulate TFEB export in response to low glucose. Indeed, activation of the feedback loop by rapamycin drove AKT phosphorylation, but unlike low glucose, it did not trigger inactivation of GSK3β. These data indicate that different triggers for AKT activation may lead to different outcomes regarding regulation of GSK3β and the TFEB export signal. In this respect, AKT has previously been implicated in regulation of TFEB, where trehalose-mediated AKT-inhibition promoted TFEB protein stability and led to increased TFEB nuclear localization in a GSK3β-independent fashion after 24 h^19^. This is clearly different from the effects of glucose deprivation on TFEB nuclear export observed here whereby inactivation of the GSK3β-dependent NES is associated with TFEB nuclear accumulation within 1 h. Moreover, although GSK3β can activate mTORC1^14^, we observe no decrease in phosphorylation of mTORC1 targets on glucose limitation, indicating that inactivation of GSK3β under these conditions is unlikely to be important in affecting mTORC1 activity. However, activation of mTORC1 signaling on glucose addition would be beneficial as it would facilitate TFEB cytoplasmic retention and we cannot rule out a role under some circumstances where GSK3β upstream of mTORC1 would play a role in TFEB regulation.

Since nuclear TFEB activates expression of genes implicated in glucose import, lipid catabolism, and glycolysis^3,17^, the inhibition of TFEB nuclear export under glucose limiting conditions will enable cells to restore carbon supply. This is likely to be especially important for the role of TFEB in promoting metabolic flexibility in exercising muscle^17^. However, TFEB nuclear export is also likely to be deregulated in diabetes where high glucose levels may diminish TFEB residence time in the nucleus thereby impairing the ability of TFEB to fine-tune the response to any intracellular supply-demand imbalance. Moreover, since nutrient limitation is also a hallmark of growing tumors and active nuclear TFEB has been observed in pancreatic cancer^22^, the coordinate regulation of the TFEB NES by phosphorylation at S142 and S138 is likely to have significant implications for the response of cancer cells to nutrient limitation within the intra-tumor microenvironment. It is also possible that the pathways regulating TFEB export may provide therapeutic opportunities for regulation of TFEB in neurodegenerative disease.

In summary, the identification of the TFEB NES and its regulation by GSK3β and mTORC2 in response to glucose availability provides a key insight into how cells coordinate the cellular response to amino acid and carbon source availability.

## Methods

### Cell culture

HT29 and MCF7 cells obtained from the ATCC were grown in a monolayer at 37 °C in humidified air containing 10% CO_2_. Cell lines were grown in Dulbecco’s Modified Eagle Medium (DMEM; Gibco) supplemented with 10% FBS. For studies of glucose starvation, 10% dialyzed FBS was substituted for 10% FBS. For live cell imaging studies, medium lacking phenol red was used. All cell lines were tested monthly for mycoplasma contamination and authenticated by Eurofins-Genomics using 21 different single locus PCR.

Transient transfection of DNA was performed using FuGENE 6 (Promega) or Lipofectamine 2000 (Fisher Scientific). Cells were transfected at 50–75% confluence in serum-free Opti-MEM (Invitrogen) according to the manufacturer’s instructions. Briefly, FuGENE 6 or Lipofectamine 2000 was incubated at room temperature in Opti-MEM for 5 min, followed by the addition of DNA and a further incubation of 15 min. The transfection mixture was then added drop-wise to cells. Cells were analyzed within the next 72 h. siRNA transfection was performed using Lipofectamine RNAiMAX (ThermoFisher Scientific) according to the manufacturer’s protocol.

For stable transfections, low-passage cells were transfected as described above with a Tet-on Piggybac expression system^46^ modified to express TFEB or derivatives, or GSK3β WT or mutants. Briefly the gene of interest, eg TFEB, is placed under the control of 6× Tet-Operators and co-transfected with a Tet-on expression vector as well as a plasmid expressing the Piggybac transposase. The transposase then integrates the expression cassette as well as the Tet-on expression vector, and polyclonal pools generated by plating cells at a confluency of 25% in appropriate selection medium. Medium was replaced every 2 days for 14 days, and cells were then screened for expression of the gene of interest in medium with or without doxycycline.

### Immunofluorescence

Cells were grown to 75% confluency on circular glass coverslips (VWR, Radnor, USA) in 24-well plates and either transfected or treated. For transfected cells, treatments were administered 18 h after transfection. For imaging, cells were fixed in 4% formaldehyde in PBS for 10 min at room temperature with agitation, followed by permeabilisation in 0.2% Triton X-100 in PBS for 7 min at room temperature with agitation. Coverslips were then blocked in 4% BSA in PBS at room temperature for 20 min with agitation. Primary antibodies were diluted 1:250 into 4% BSA in PBS and added to coverslips for 20 min at room temperature upside down on PARAFILM M (Sigma-Aldrich). After washing three times in PBS for 3 min each, secondary antibodies were added (1:500) in a solution of 4% BSA in PBS for 20 min at room temperature upside down on PARAFILM M in the dark. DAPI was added to the secondary antibody solution to stain nuclei (1:2000). Coverslips were then mounted on glass microscope slides (VWR) with Vectorshield H-1000 mounting medium (Vector laboratories). A Zeiss LSM710 and accompanying Zen software were used to acquire confocal images (Zeiss, Oberkochen, Germany). Images were processed for presentation using Photoshop. Antibodies used are listed in Supplementary Table 2.

### Fluorescence live cell imaging

Cells were plated in 6- or 12-well plates and either transfected or treated. Plates were then placed on the stage of a Nikon TE 2000-E Eclipse inverted microscope in a climate-controlled (37 °C; 10% CO_2_) incubation chamber. After being allowed to acclimate for 1 h, images were acquired with a Hamamatsu Orca-ER C4742-95 camera and Metamorph software (Molecular Devices, Sunnyvale, USA). If a timelapse series was needed, this image served as the initial timepoint, with subsequent imaging being automated via the Metamorph software. The multidimensional acquisition option was used to capture DAPI, GFP and RFP channels as required, with the system set to autofocus for every exposure.

If the experimental design required altering the culture medium during the course of the experiment, the system was paused. Where medium was exchanged for medium lacking a factor, the existing medium was aspirated, cells rinsed once with the medium to be added, the new medium added and the plate replaced on the stage. Where a factor was added or replenished, as in experiments involving addition of inhibitors or replenishment of nutrients, the lid of the plate was removed and the plate left in position on the stage. Medium containing the factor at 10× the final concentration was then carefully added dropwise to the medium. The lid was then replaced, and imaging was resumed. Images were then processed in ImageJ. To visualize nuclei, cells were incubated with Hoechst 33342 (100 ng) for 30 min prior to treatment with Torin or glucose free medium (including 10% dialyzed FBS).

### Subcellular fractionation

To obtain nuclear and cytoplasmic fractions, an adaptation of the Rapid Efficient And Practical method was used (REAP)^47^. Briefly, cells were rinsed with cold PBS and scraped in 500 µl of 0.1% NP-40 in PBS. One third of the sample was taken as a whole cell lysate, while the remainder was centrifuged at 13,000×*g* for 30 s. From the supernatant, 150 µl was taken as a cytoplasmic fraction, while the remainder was discarded. The pellet was then washed with 1 ml of 0.1% NP-40 in PBS. After centrifugation at 13,000 g for 30 s, the supernatant was discarded. The pellet was resuspended in 1× Laemmli buffer and processed as the nuclear fraction.

### SDS PAGE and western blotting

Whole cell extracts were prepared by the direct addition of 1× Laemmli sample buffer (62.5 mM Tris [pH 6.8], 2% SDS, 10% glycerol, 0.02% bromophenol blue, 5% 2-mercaptoethanol) to the cells in the culture vessel. Cells were scraped with a cell scraper (TPP, Trasadingen, Switzerland), and lysates were collected and sonicated twice for 3 s with a probe sonicator (Sonics, Newton, USA). Where samples were prepared by some other means, 2× or 4× Laemmli sample buffer was added to yield a final concentration equating to 1× Laemmli sample buffer. Samples were then incubated at 95 °C for 5 min and stored at −20 °C until use. Cell extracts were analyzed by SDS polyacrylamide gel electrophoresis (SDS-PAGE) and Western blotting using 12% polyacrylamide gels (37.5:1 acrylamide:bis-acrylamide; Severn Biotech, Kidderminster, UK). Gels were run at 150 V for 60–90 min. The protein ladder Precision Plus Protein All Blue Protein Standard (Bio-Rad) was used to indicate molecular weight. Proteins were transferred onto a Protran nitrocellulose membrane (Whatman, Kent, UK) by electroblotting at 150 mA for 90 min on ice in a transfer buffer comprising 25 mM Tris, 192 mM glycine and 20% methanol. Ponceau staining (0.1% Ponceau S in 5% acetic acid; 5 min) was used to assess the efficacy of the transfer and to facilitate cutting the membrane when probing the same membrane with multiple antibodies.

After rinsing off the Ponceau staining solution, membranes were incubated with agitation at room temperature for 1 h in 5% bovine serum albumin (BSA) in 0.1% Tween in TBS (TBST). Primary antibodies were diluted into 5% BSA in TBST and incubated with membranes overnight at 4 °C with agitation. Membranes were then washed 3× in TBST and incubated with the appropriate horseradish peroxidase (HRP)-conjugated secondary antibody (Bio-Rad) diluted 1:10,000 in 5% BSA in TBST for 1 h at room temperature with agitation. After 3 washes in TBST, membranes were incubated with enhanced chemiluminescence (ECL) reagent (Amersham, Uppsala, Sweden). X-ray film (Fujifilm, Tokyo, Japan) was exposed to the membranes and developed in a dark room. Antibodies used are listed in Supplementary Table 2.

### qRT-PCR analysis

RNAs were extracted using RNeasy mini kit (Qiagen) following manufacturer’s instructions. cDNA was synthesized using QuantiTect Reverse Transcritpion Kit (Qiagen), and Brilliant III Ultra-Fast SYBR Green QPCR Master Mix (Agilent Technologies) was used in the qRT-PCR reaction. qRT-PCR reactions were performed using Rotor-gene Q or Rotor-gene 6000 machines (Qiagen). Primer sequences used for qRT-PCR can be found in Supplementary Table 3. Statistics were performed using the delta CT method.

### Site directed mutagenesis

Mutations were introduced using the QuikChange Lightning Site-Directed Mutagenesis Kit (Agilent, Santa Clara, USA) using primers designed with the QuikChange Primer Design Program (http://www.genomics.agilent.com). Reactions used 20 ng of plasmid DNA, 125 ng of each primer, 1 µl dNTP mix, 1.5 µl QuikSolution, 5 µl 10× buffer, and 1 µl QuikChange Lightning enzyme in a total volume of 50 µl. Samples were then incubated in a T100 Thermal Cycler using the following program: 95 °C denaturation for 2 min, followed by 25 cycles of denaturation (95 °C; 15 s), annealing (58 °C; 15 s) and extension (68 °C; 30 s/kb of plasmid), and a final extension step (68 °C; 10 m). Samples were then incubated with 2 µl DpnI at 37 °C for 1 h, followed by transformation into XL10-Gold ultracompetent cells (Agilent). Sanger sequencing was used to screen colonies for successful insertion of point mutations. Oligonucleotides used for mutagenesis are listed Supplementary Table 3.

### Peptide SPOT kinase assay

Peptides 19 amino acids long were synthesized on cellulose membranes using SPOT technology^48^. Membranes were rinsed with 95% ethanol, followed by washing 5× with TBST. GSK3 kinase (NEB) was then prepared in a reaction mixture comprising 50 ng of kinase, 200 μM ATP (Sigma-Aldrich) and 8 µCi [γ-^32^P]-ATP (Perkin Elmer) in 1.5 ml of 1× NEBuffer for Protein Kinases (NEB). The reaction mixture was then placed on the membrane, which was incubated at 30 °C for 30 min. The membrane was then washed 15× with NaCl (1 M), followed by washing 10× with water before washing with stripping buffer (1% SDS, 8 M urea, 0.5% 2-mercaptoethanol) for 1 h at 40 °C. This was followed by washing 10× in water and a final wash in 95% ethanol. After air drying the membrane for 10 min, it was exposed to a phosphor screen overnight for 3 h. Images were acquired with a PMI phosphorimager (Bio-Rad).

### Anti-pS142 validation

A peptide array synthesized on a cellulose membranes was re-hydrated by rinsing three times with 100% ethanol and then equilibrated by washing three times for 5 min in PBS-T. After, the membrane was blocked in 5% BSA PBS-T (pH 7.4, 11.9 mM phosphates, 137 mM NaCl, 2.7 mM KCl with 0.1% Tween-20) for 8 h at room temperature. The membrane was then washed three times for 5 min with PBS-T before incubating with the anti-pS142 rabbit antibody (Sigma Aldrich, Cat. No. SAB4503940) diluted 1:5,000 in 5% BSA 1XPBS-T overnight at 4 °C, shaking. The next morning, the membrane was washed three times for 5 min in 1XPBS-T before re-blocking in 5% BSA 1XPBS-T at room temperature for 1 h, shaking. After, the membrane was washed three times for 5 min in 1XPBS-T before incubating with the HRP-conjugated secondary antibody (Bio-Rad) diluted 1:10,000 in 5% BSA 1XPBS-T for 1 h, at room temperature, shaking. The membrane was washed three times for 20 min with PBS-T at room temperature, shaking before incubating with ECL reagent (Amersham, Uppsala, Sweden). Excess ECL reagent was removed before imaging with the ImageQuant Las-4000 camera. The results of the spot kinase assay are shown in Supplementary Figure 1.

### Bacterial expression of TFEB and in vitro phosphorylation

*E. coli* (Rosetta strain) transformed with the pNIC TFEB 1–200 6× nHis expression vector and selected using Kanamycin (50 μg mL^−1^) were amplified at 37 °C in LB medium containing Kanamycin (50 μg mL^−1^) and Chloramphenicol (34 μg mL^−1^). Once an OD_600_ of 0.6 was reached, the culture was cooled to 18 °C and recombinant TFEB expression induced by adding 0.1 mM IPTG and incubating overnight at 18 °C. Cells were harvested by centrifugation at 6,000×*g* for 15 min before lysing using denaturing conditions (6 M urea, 25 mM HEPEs pH 7.5, 0.5 M NaCl, 5% Glycerol, 20 mM Imidazole, buffer pH 7.5) using a French Press homogenizer. The lysate was then cleared by centrifugation at 25,000×*g* for 15 min. 10 mL Ni-NTA agarose (QIAGEN, Cat No. 30210) was washed twice using 10 mL Lysis Buffer before cleared lysate was added to the resin and incubated at 4 °C, rotating for 1 h. The lysate-resin mixture was applied to a gravity-flow column before washing twice with 10 mL wash buffer (6 M urea, 25 mM HEPEs pH 7.5, 0.5 M NaCl, 5% Glycerol, Buffer pH 7.5) and once with Lysis Buffer. Protein was eluted using increasing concentrations of Imidazole (50–250 mM). Eluted TFEB was visualized by SDS-PAGE (12% polyacrylamide, ran at 150 V, 1 h) using the Instant blue Coomassie staining reagent (Expedeon, Cat No. ISB1L). Protein from eluted fractions were pooled and dialyzed using 3–5 kDa DiaEasy Dialyzer tubes (Biovision, Cat No. K1012) using buffers prepared with decreasing concentrations of urea (25 mM HEPEs pH 7.5, 0.5 M NaCl, 5% Glycerol, 4.0-0.0 M Urea). Protein fractions were dialyzed for 1.5 h each urea step at 4 °C on a magnetic stirrer starting with 1 L of 4 M urea (two times), then 2 M urea, and then 1 M urea before transferring to 0.5 M urea at 4 °C on a magnetic stirrer overnight. The next morning, samples were dialyzed against 1 L of urea-free buffer for 3.0 h at 4 °C on a magnetic stirrer. Once dialysis was complete, samples were aliquoted, flash frozen in liquid nitrogen before transfer to −80 °C for long-term storage.

For in vitro kinase assays, reaction mixes were prepared using 2 μg of recombinant TFEB (1–200 6xnHis), 0.3 mM cold ATP and 100 ng of ERK2 (Sigma Aldrich, Cat No. E1283-10UG) and/or 100 ng of GSK3β (Abcam, Cat No. ab60863). All reactions were made up to 22 μl with Kinase Dilution Buffer (KDB). KDB was prepared by diluting Kinase Assay Buffer (25 mM MOPS pH 7.2, 12.5 mM glycerol 2-phosphate, 25 mM MgCl_2_, 5 mM EGTA, and 2 mM EDTA) 1:5 in ddH_2_0 before adding DTT (0.25 mM). Reactions were incubated for a total of 1 h at 37 °C. ERK2 was added at the beginning of the experiment, whereas GSK3β was added after 30 min (kept consistent among all samples). Afterwards, 100 mM EDTA was added to chelate Mg^2+^ ions in order to prevent further kinase activity.

### Mass spectrometry analysis

Following in vitro phosphorylation, proteins were digested using SMART digest (Thermo Fisher) according to the manufacturer’s instructions. Samples were desalted using SepPak reversed phase columns and injected into an LC-MS/MS platform consisting of Dionex Ultimate 3000 nano LC and Orbitrap Fusion Lumos instruments. Sample separation was achieved with a 60 min gradient of 2–35% acetonitrile in 0.1% formic acid and 5% DMSO and 250 nl/min flow rate with an EasySpray column (ES803, Thermo Fisher) of 50 cm length and 75 µm inner diameter. MS1 spectra were acquired with a resolution of 120.000 and an ion target of 400.000, followed by a top speed duty cycle of up to 3 s for MS/MS acquisition. Precursor ions were isolated in the quadrupole with a mass window of 1.6 Th and fragmented with HCD@28% normalized collision energy. MS/MS data was acquired in the ion trap with rapid scan mode and an ion target of 4000.

LC-MS/MS data was analyzed using PEAKS Server V.7 (Bioinformatics Solutions) and a Uniprot/Trembl database. Variable modifications were set to Phosphorylation (S, T, Y), Oxidation (M) and Deamidation (N, Q). Mass tolerance was 10 ppm for precursor and 0.5 Da for fragment mass. The false discovery rate was set to 1% at peptide level. Extracted ion chromatograms of relevant peptides were generated with Freestyle 1.3 (Thermo Fisher) and quantified after Gaussian smoothing (3 data points).

### Bacterial expression of RAN and CRM1 and in vitro binding

Human CRM1 was overexpressed in the BL21(DE3) strain of *Escherichia coli* with an N-terminal GST-tag. When the OD at 600 nm of the bacterial culture reached 1.0, recombinant CRM1 expression was induced with 0.5 mM isopropyl-β-d-thiogalactopyranoside (IPTG) and allowed to grow at 18 °C overnight. The cells were harvested and resuspended in TB buffer [50 mM Tris (pH 7.5), 200 mM NaCl, 10% (vol/vol) glycerol, 1 mM EGTA, 5 mM DTT] supplemented with protease inhibitors (Roche). The cells were lysed by sonication in 20 mL L^−1^ of culture and clarified by centrifugation at 50,000×*g* (30 min at 4 °C). The supernatant was bound to preequilibrated glutathione Sepharose 4B (GE Healthcare) for 1 h by rotating at 4 °C. After extensive washing, the bound GST-CRM1 was cleaved with TEV protease. CRM1 was then eluted and loaded onto a Mono Q HP (GE Healthcare) column. The protein was eluted with a salt gradient ranging from 0 to 1 M NaCl in 20 mM Tris (pH 7.5), 10% (vol/vol) glycerol, 2 mM magnesium acetate, 1 mM EGTA, and 1 mM DTT.

Human Ran protein (pET15b) was induced with 0.5 mM IPTG and allowed to grow at 20 °C for 18 h. The cells were harvested and resuspended in 20 mL L^−1^ of cold lysis buffer [20 mM Tris (pH 8.0), 100 mM NaCl, 10% (vol/vol) glycerol, 1 mM β-mercaptoethanol, 10 mM Imidazole, 1 mg mL^−1^ lysozyme, protease inhibitors]. After sonication, the cleared lysate was purified by affinity chromatography using Ni-NTA beads. After a washing step with buffer containing 50 mM Imidazole, the protein was eluted with 500 mM imidazole and further separated using gel filtration chromatography (Superdex-75 column; Amersham Biosciences) in 20 mM Tris (pH 8.0), 10% (vol/vol) glycerol, and 1 mM DTT. The GTP-bound form was obtained by incubating Ran with 1,000 molar-excess of GTP in the presence of 50 mM Hepes pH 7.5, 10 mM EDTA, 2 mM ATP, and 4 mM DTT for 30 min at 30 °C. The reaction was stopped at 4 °C by adding 15 mM MgCl_2_.

For the CRM1 pull down assays TFEB (aas 1–200) was expressed in *E. coli* BL21(DE3) cells (grown to an OD of 0.6–0.8 and then induced overnight with 0.1 mM IPTG at 18 °C). The cells were lysed by sonication in denaturing buffer containing 50 mM Hepes (pH 7.5), 500 mM NaCl, 20 mM Imidazole, 5% (vol/vol) glycerol, 10 mM β-mercaptoethanol, and 6 M urea. Protein was purified by affinity chromatography using Ni-NTA beads. After extensive washing, protein was refolded on the column by slowly decreasing the urea concentration from 6 M to 0 M urea (6 M, 4 M, 2 M, 1 M, 0.5 M, 0.2 M, and 0 M). A gradient volume of 10 mL and a flow rate of 1 mL/min were used. 100 pmol of bound TFEB was used for each binding reaction, with 50 pmol of purified CRM1 in the presence or absence of twofold molar excess of Ran-GTP. The binding was performed in 400 μL of binding buffer [20 mM Hepes (pH 7.5), 110 mM potassium acetate, 2 mM magnesium acetate, 0.01% Nonidet P-40, 1 mM EGTA, 20 mM DTT, 20 mM Imidazole] and mixed by rotation for 2 h at 4 °C. Unbound protein was removed by washing four times with binding buffer, and bound proteins were eluted with hot Laemmli sample buffer. The eluted protein complexes were then separated by SDS/PAGE and CRM1 was observed by Western blot.

### Plasmids

Details of plasmids used are listed in Supplementary Table 4 and are available on request.

### Statistical analysis and original data

For all quantifications of fluorescent images mean±s.d. were derived from the number of experiments or cells and analyzed by *t*-test or ANOVA. See Supplementary Table 1 for details. *p < 0.05, **p < 0.01, ***p < 0.001, ****p < 0.0001. Statistical analysis was performed using GraphPad Prism 7. Western blots presented as representative images derived from multiple successful experiments in which reproducibility was ensured by using alternative approaches in independent experiments in different cell lines and by using, where appropriate, alternative antibodies.

### Data availability

We declare that all data supporting the findings of this study are available within the article or from the corresponding author upon request. Source data for all western blots and the Coomassie gels is shown in Supplementary Figure 2. Mass Spectrometry data was deposited at the ProteomeXchange Consortium via the PRIDE partner repository with the dataset identifier [PXD009677].

## Electronic supplementary material

Supplementary Information

Description of Additional Supplementary Files

Supplementary Movie 1
