# Supplementary material for: A TFEB nuclear export signal integrates amino acid supply and glucose availability
Source: Nat Commun. 2018 Jul 11;9:2685. doi: 10.1038/s41467-018-04849-7 (PMC6041281; doi:10.1038/s41467-018-04849-7)
Supplement: Supplementary file 1 — Supplementary Information [file 41467_2018_4849_MOESM1_ESM.pdf]

Supplementary Information

A TFEB nuclear export signal integrates amino acid supply and glucose availability

Li et al

Supplementary Figure 1
Supplementary Figure 2
Supplementary Table 1
Supplementary Table 2
Supplementary Table 3
Supplementary Table 4

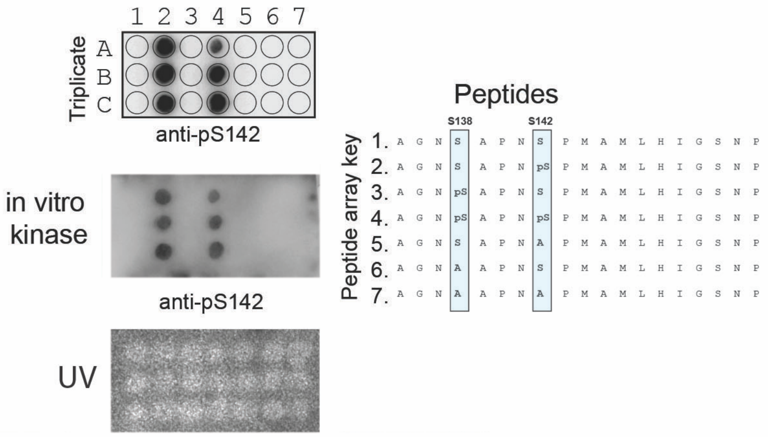

Supplementary Figure 1
Validation of commercial antibody (Sigma-Adrich SAB4503940) recognising TFEB phospho-S142 using indicated peptide dot blot

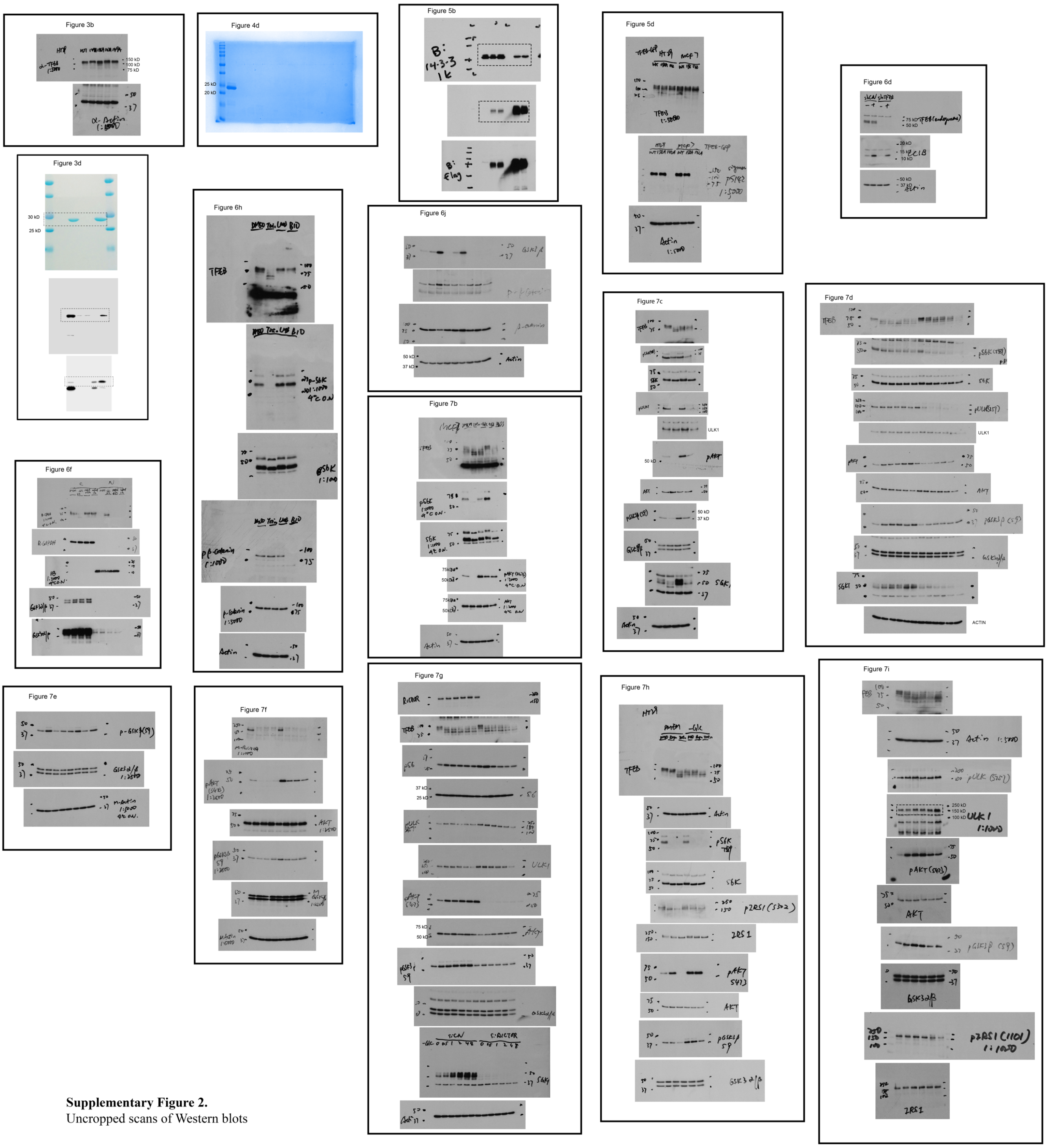

Supplementary Figure 2.
Uncropped scans of Western blots

Supplementary Table 1.

Summary for quantification of fluorescent images

	n (Number of Cells/Lysosomes)	Number of Technical Replicates	Number of Biological Replicates	Statistical Test
Figure 1 a	113	3~4	1	t test
Figure 1 c	114	3	1	t test
Figure 1 d	35	4~5	1	t test
Figure 1 e (left)	160	4~5	2	t test
Figure 1 e(right)	9596	3	3	t test
Figure 2 a	288	2	1	t test
Figure 2 c	3952	3	2	t test
Figure 3 a	4153	3	2	t test
Figure 3 c	14120	3	3	t test
Figure 3 f	1484	10	3	t test
Figure 4 a	1740	3	2	t test
Figure 5 a	4874	3	3	t test
Figure 5 c	1433	5~10	3	t test
Figure 5 e	1038	3~5	4	t test
Figure 6 a	2202	1	5	t test
Figure 6 b	926	3~6	2	t test
Figure 6 e	1151	2	3	t test
Figure 6 g	36516	3	3	t test
Figure 6 i	9132	5	3	t test
Figure 6 k	6081	5~10	3	t test

Supplementary Table 2
Summary of antibodies used

Antibody	Immunogen	Source (Catalogue)	Dilution (Application)
α-actin (AC-40) mouse monoclonal	C-terminus	Sigma-Aldrich A4700	1:1000 (WB)
α-HA (HA-7) mouse monoclonal	Residues 98-106 (YPYDVPDYA) of human Influenza virus hemagglutinin (HA)	Sigma-Aldrich H3663	1:500 (IF)
α-ERK (C-14) rabbit polyclonal	C-terminus	Santa Cruz sc-154	1:10000 (WB)
α-GAPDH rabbit polyclonal	Residues 314-333	Sigma-Aldrich G9545	1:20000 (WB)
α-GFP rabbit polyclonal	Full-length GFP	Abcam ab290	1:20000 (WB) 1:1000 (IF)
α-H3 rabbit polyclonal	Residue 100 to C-terminus	Abcam ab1791	1:2,000 (WB)
α-p70 S6 Kinase (C-18) rabbit polyclonal	C-terminus	Santa Cruz sc-230	1:1000 (WB)
α-S6 Ribosomal Protein (5G10) rabbit monoclonal	Residues of human S6 ribosomal protein	Cell Signaling 2217	1:1000 (WB)
α-phospho ERK1/2 (T202/Y204) rabbit polyclonal	Region surrounding T202/Y204	Cell Signaling 4377	1:1000 (WB)
α-phospho p70 S6 Kinase (T389) rabbit polyclonal	Region around T389	Cell Signaling 9205	1:1000 (WB)
α-phospho-S6 Ribosomal Protein (Ser235/236) rabbit polyclonal	Residues surrounding Ser235 and Ser236 of human ribosomal protein S6	Cell Signaling 2211	1:1000 (WB)
α-TFEB rabbit polyclonal	Region surrounding G412	Cell Signaling 4240	1:1000 (WB) 1:250 (IF)
α-phospho-Akt (Ser473) rabbit polyclonal	Residues surrounding Ser473 of mouse Akt	Cell Signaling 9271	1:2000 (WB)
α-Akt rabbit polyclonal	C-terminal of of mouse Akt	Cell Signaling 9272	1:2000 (WB)
α-phospho-GSK3β (S9) rabbit polyclonal	Residues surrounding S9 of human GSK3	Cell Signaling 9336	1:2000 (WB)
α-GSK3α/β (21A) mouse monoclonal	Full length *Xenopus laevis* GSK3	Invitrogen 44-610	1:2000 (WB)
α-RICTOR (7B3) mouse monoclonal	Full length Rictor	Abnova MAB10313	1:1000(WB) 1:100 (IF)
α-phospho-IRS-1 (S302) rabbit polyclonal	Residues surrounding Ser 302 of mouse IRS-1	Cell Signaling 2384	1:1000 (WB)

α-phospho-IRS-1 (S1101)<br>rabbit polyclonal	Residues surrounding Ser1101 of human IRS-1	Cell Signaling 2385	1:1000 (WB)
α-IRS-1<br>rabbit polyclonal	Carboxy-terminal sequence of human IRS-1	Cell Signaling 2382	1:1000 (WB)
α-phospho-ULK1 (S757)<br>rabbit polyclonal	Residues surrounding Ser757 of mouse ULK1	Cell Signaling 6888	1:1000 (WB)
α-ULK1 (D8H5)<br>rabbit monoclonal	Residues surrounding Arg600 of human ULK1	Cell Signaling 8054	1:1000 (WB)
α-SGK1<br>rabbit polyclonal	Residues surrounding Gly420 of human SGK1	Cell Signaling 12103	1:1000 (WB)
α-LC3B<br>rabbit polyclonal	Residues near the amino terminus of LC3B	Cell Signaling #2775	1:1000 (WB)
α-phospho-MITF (pSer130/73)/ phospho-TFEB (Ser142)<br>Rabbit polyclonal	Synthesized peptide derived from human MITF around the phosphorylation site of Ser180/73	Sigma-Aldrich SAB4503940	1:5000 (WB)
α-RAN<br>Mouse monoclonal	Human Ran aa. 7-171	BD Biosciences #610341	1:1000 (WB)
α-CRM1<br>Goat plolyclonal	C-terminal 20 aa of human CRM1	Santa Cruz # sc-7825	1:2000 (WB)

Supplementary Table 3
Summary of oligonucleotides used

All oligonucleotides were made by Integrated DNA Technologies (Leuven, Belgium).

**Use**	**Details**	**Sequence**
Cloning	Clone human TFEB residues 1-159 into nlsCARGO, AgeI, forward	CTAGACCGGTGCCACCATGGCGTC ACGCATAGGGTTGCG
Cloning	Clone human TFEB residues 1-159 into nlsCARGO, AgeI, reverse	CTAGACCGGTATATCATCCAACTC CCTCTCAGGGTTGG
Cloning	Clone human TFEB residues 129-152 into nlsCARGO, EcoRI, forward	CTAGGAATTCACACGTGCTGTCCT CCTCCGCTG
Cloning	Clone human TFEB residues 129-152 into nlsCARGO, KpnI, reverse	CTAGGGTACCGTTGGAGCCAATGT GCAGCATGG
Cloning	Clone TFEB-GFP into pPB-iCMV-MCS, SacII, forward	CTAGCCGCGGGCCACCATGGCGTC ACGC
Cloning	Clone TFEB-GFP into pPB-iCMV-MCS, NotI, reverse	CTAGGCGGCCGCTCACAGCACATC GCCC
Cloning	Clone 3xHA-GSK3β into pPB-iCMV-MCS, EcoRI, forward	GCTAGAATTCGCCACCATGTCAGG GCGGCCCAG
Cloning	Clone 3xHA-GSK3β into pPB-iCMV-MCS, NotI, reverse	GCTAGCGGCCGCCTAGTTGGGACC ACCCAAGCT
Site-directed mutagenesis	TFEB S138A mutagenesis forward	CCTCCGCTGGCAACGCTGCTCCCA ATAGCC
Site-directed mutagenesis	TFEB S138A mutagenesis reverse	GGCTATTGGGAGCAGCGTTGCCAG CGGAGG
Site-directed mutagenesis	TFEB S142A mutagenesis forward	TGGCAACAGTGCTCCCAATGCCCC CATG
Site-directed mutagenesis	TFEB S142A mutagenesis reverse	GGCCATGGGGGCATTGGGAGCACT GTTG
Site-directed mutagenesis	TFEB M144A mutagenesis forward	GCTCCCAATAGCCCCGCGGCCATG CTGCACAT
Site-directed mutagenesis	TFEB M144A mutagenesis reverse	ATGTGCAGCATGGCCGCGGGGCTA TTGGGAGC
Site-directed mutagenesis	TFEB M146A mutagenesis forward	CAATAGCCCCATGGCCGCGCTGCA CATTGGCTCC
Site-directed mutagenesis	TFEB M146A mutagenesis reverse	GGAGCCAATGTGCAGCGCGGCCAT GGGGCTATTG
Site-directed mutagenesis	TFEB L147A mutagenesis forward	GCCCCATGGCCATGGCGCACATTG GCTCCA
Site-directed mutagenesis	TFEB L147A mutagenesis reverse	TGGAGCCAATGTGCGCCATGGCCA TGGGGC
Site-directed mutagenesis	TFEB I149A mutagenesis forward	CATGGCCATGCTGCACGCTGGCTC CAACCCTGAG
Site-directed mutagenesis	TFEB I149A mutagenesis reverse	CTCAGGGTTGGAGCCAGCGTGCAG CATGGCCATG
Site-directed mutagenesis	TFEB S211A mutagenesis forward	GTCACCAGCAGCGCCTGCCCTGCG G
Site-directed mutagenesis	TFEB S211A mutagenesis reverse	CCGCAGGGCAGGCGCTGCTGGTGA C

RT-PCR analysis	Human TFEB gene, forward	CAAGGCCAATGACCTGGAC
RT-PCR analysis	Human TFEB gene, reverse	AGCTCCCTGGACTTTTGCAG
RT-PCR analysis	Human GAPDH gene, forward	TGCACCACCAACTGCTTAGC
RT-PCR analysis	Human GAPDH gene, reverse	TCTTCTGGGTGGCAGTGATG
RT-PCR analysis	Human *CDKN1A* gene, forward	GGCAGACCAGCATGACAGATT
RT-PCR analysis	Human *CDKN1A* gene, reverse	GCGGATTAGGGCTTCCTCTT
RT-PCR analysis	Human *RRAGD* gene, forward	GTGACCAGGGCCTACAAAG
RT-PCR analysis	Human *RRAGD* gene, reverse	TTTGCCCTCTGGTGAATATCT

Supplementary Table 4

Summary of plasmids used

Details of construction of all plasmids used are available on request

Construct	Comments
pEGFP-C1-TFEB	Addgene; GFP-tagged human TFEB
pEGFP-C1-TFEB(S211A)	TFEB(S211A)-GFP
pEGFP-C1-TFEB(S142A)	TFEB(S142A-GFP
pEGFP-C1-NLS-GST	GFP-NLS-GST cargo (nlsCARGO)
pEGFP-C1-NLS-GST-NES	nlsCARGO with PKI NES (nlsCARGO-NES)
pEGFP-C1-NLS-GST-TFEB(1-159)	nlsCARGO with TFEB residues 1-159
pEGFP-C1-NLS-GST-TFEB(129-152)	nlsCARGO with TFEB residues 129-152
pEGFP-C1-NLS-GST-TFEB(1-159)(S142A)	TFEB(1-159)(S142A)-nlsCARGO
pEGFP-C1-NLS-GST-TFEB(129-152)(S142A)	TFEB(129-152)(S142A)-nlsCARGO
pPB-iCMV-MCS	PiggyBac vector with Tet-On inducible system
pPB-iCMV-TFEB-GFP	TFEB-GFP in Tet-On with piggyBac transposon elements
pPB-iCMV-TFEB(S138A)-GFP	TFEB(S138A)-GFP in Tet-On with PiggyBac transposon elements
pPB-iCMV-TFEB(S142A)-GFP	TFEB(S142A)-GFP in Tet-On with PiggyBac transposon elements
pPB-iCMV-TFEB(M144A)-GFP	TFEB(M144A)-GFP in Tet-On with PiggyBac transposon elements
pPB-iCMV-TFEB(M146A)-GFP	TFEB(M146A)-GFP in Tet-On with PiggyBac transposon elements
pPB-iCMV-TFEB(L147A)-GFP	TFEB(L147A)-GFP in Tet-On with PiggyBac transposon elements
pPB-iCMV-TFEB(I149A)-GFP	TFEB(I149A)-GFP in Tet-On with PiggyBac transposon elements
pPB-iCMV-TFEB(S211A)-GFP	TFEB(S211A)-GFP in Tet-On with PiggyBac transposon elements
pPB-iCMV-3xHA-GSK3β (S9A)	3xHA-GSK3β (S9A)in Tet-On with PiggyBac transposon elements
pPB-iCMV-3xHA-GSK3β (K85A)	3xHA-GSK3β (K85A)in Tet-On with PiggyBac transposon elements
pmCherry-C1-RanQ69L	Addgene #30309, a gift from Jay Brenman
pmCherry-LacR-NLS	[26] Soutoglou, E. & Misteli
