# Supplementary material for: A TFEB nuclear export signal integrates amino acid supply and glucose availability
Source: Nat Commun. 2018 Jul 11;9:2685. doi: 10.1038/s41467-018-04849-7 (PMC6041281; doi:10.1038/s41467-018-04849-7)
Supplement: Supplementary file 2 — Description of Additional Supplementary Files [file 41467_2018_4849_MOESM2_ESM.pdf]

Description of Additional Supplementary Files

File Name: Supplementary Movie 1
Description: Real time imaging of MCF7 iTFEB-GFP cells over 20 min. Cells corresponding to those shown in **Fig. 1b** are found in the bottom left corner.
